# Design of an implementation research study for a digital omnichannel community engagement and risk communication intervention for the prevention and control of vector-borne diseases in India: the OMNIVEC-India study protocol

**DOI:** 10.3389/fpubh.2025.1710603

**Published:** 2026-02-16

**Authors:** Rashmi Rodrigues, Amey Dhatrak, Madhusmita Bal, Melari Shisha Nongrum, Neeraj Kumar, Rajiv Sarkar, Twinkle Agrawal, Anuj Mundra, Ira Praharaj, Jyoti Singh, Mrunali Zode, Sudipto Roy, Vani Kandpal, Tanica Lyngdoh, Omnivec Study Team

**Affiliations:** 1 St John's Medical College, Bengaluru, India; 2 Mahatma Gandhi Institute of Medical Sciences, Sevagram, India; 3 ICMR - Regional Medical Research Centre Bhubaneswar, Bhubaneswar, India; 4 Indian Institute of Public Health, Shillong, India; 5 ICMR-National Institute of Child Health and Development Research, New Delhi, India; 6 Indian Council of Medical Research, New Delhi, India; 7 Faculty of Medical Research, Academy of Scientific and Innovative Research (AcSIR), Uttar Pradesh, India; 8 Faculty of Biological Sciences, Academy of Scientific and Innovative Research (AcSIR), Uttar Pradesh, India

**Keywords:** vector borne disease, dengue, filariasis, malaria, risk communication and community engagement, digital communication, social and behavioral change communication, India

## Abstract

**Introduction:**

Vector-borne diseases (VBDs), such as malaria, dengue, and lymphatic filariasis, pose significant public health challenges in India, contributing significantly to the nation’s morbidity and mortality. In this scenario, digital technology promises scalable, contextual, and user-centric interventions for behavior change. However, systematic and comprehensive community-based digital approaches for VBD prevention in India are limited. This study, therefore, aims to co-design a digital omnichannel Community Engagement and Risk Communication (CERC) intervention for VBDs in collaboration with the community and the health system, and pilot the intervention for acceptability and effectiveness.

**Methods and analysis:**

This mixed-methods implementation research will be conducted across five Indian states: Punjab, Maharashtra, Karnataka, Odisha, and Meghalaya, over 2 years. The initial formative phase will involve quantitative surveys to assess community knowledge, attitudes, practices, access to digital media, and perceptions of vector-borne disease risk. Qualitative studies will include participatory appraisals, stakeholder mapping, focus group discussions, and in-depth interviews. Additionally, a review of existing global community engagement and risk communication models is planned. Organizational readiness for digital CERC among public health stakeholders will also be evaluated. The subsequent Development Phase will focus on the collaborative creation and early implementation of a context-specific digital intervention delivered through multiple channels, guided by the EPIS framework. The implementation outcomes will include the intervention’s acceptability, feasibility, fidelity, and early adoption. A combination of qualitative and quantitative methods, along with internet metrics such as calls to action (CTA), will support the evaluation.

**Impact:**

A co-designed VBD-CERC strategy that ensures community/user-centricity is likely acceptable and sustainable within local communities. Such a strategy is also likely to bring about the desired public health outcomes. While this intervention primarily targets VBDs, it also has potential applications in other diseases, both in India and globally.

## Introduction

Vector-borne diseases (VBDs) such as malaria, dengue, chikungunya, leishmaniasis, and lymphatic filariasis (LF) affect nearly half of the global population (1). VBDs account for >17% of all infectious diseases globally, approximately 0.7 million deaths, and 0.8–46 million disability adjusted-life years based on the disease annually (1). VBDs are an eco-bio-social phenomenon involving the environment, the vector, and humans, including their behavior (2). The environment, especially climate change, coupled with the tropical climate in countries like India, is conducive to vector proliferation and VBD endemicity (3). Additionally, urbanization and socio-demographic transitions have also contributed to the changing epidemiological trends in VBDs (4).

While ticks, mites, lice, and mosquitoes are involved in VBD transmission, mosquitoes are the most commonly implicated. Mosquito-borne VBDs such as malaria, dengue, LF, Japanese encephalitis, and chikungunya are endemic in several parts of India (5). Of these, malaria, dengue, and lymphatic filariasis contribute the most to morbidity, mortality, and economic burden, requiring urgent public health action (5).

Despite decades of national health programs aimed at prevention, India continues to disproportionately bear the burden of VBDs globally. While Southeast Asia accounts for only 2% of the global burden of malaria, India alone accounts for 82% of the malaria cases within this region (6). Additionally, India contributes 40% to the global burden of LF (7) and 34% to the global burden of symptomatic dengue infections (8). Furthermore, India has witnessed a tenfold increase in the burden of dengue since the year 2000, causing an estimated 40,000 deaths annually (8). This study, therefore, prioritizes dengue and malaria due to their high prevalence and public health impact and includes LF, as it remains a nationally prioritized disease targeted for elimination.

In this scenario, Community Engagement and Risk Communication (CERC) strategies are foundational to VBD prevention and control. Community engagement (CE) helps contextualize health interventions, ensuring local ownership and uptake, while risk communication (RC) enables timely and accurate dissemination of messages targeting people likely to acquire an illness of public health concern (9). CERC strategies aimed at supporting sustainable and resilient VBD programs are therefore the crux of the Global Vector Control Response 2017–2030 (1).

Several countries have successfully used CERC to eliminate VBDs. For instance, community ownership models in Thailand, Indonesia, and the Philippines significantly reduced malaria and LF transmission (10). India has also demonstrated an 80% reduction in the burden of malaria in Odisha through ASHA-led malaria screening (DAMaN) (11, 12), while intensive community engagement and mobilization were used to reduce vector breeding in Tamil Nadu and Kerala (13, 14).

Digital technology has shrunk the world. It has revolutionized communication, especially health communication, both globally and in India (15). Given that India has 1.2 billion mobile phone subscribers, of whom 70% use smartphones, digital technology offers scalable, cost-effective alternatives for health communication, especially for underserved and hard-to-reach populations (16).

Given this scenario, the use of digital technology within national programs, such as the National Vector Borne Disease Control Program (NVBDCP), which currently rely on one-way communication campaigns, has immense scope to enhance interactivity and continuity (17). We therefore propose to develop and test a digital omnichannel health intervention that harnesses social media and the Internet and supplements these with offline communication for CERC in VBDs.

The intervention is based on the transtheoretical model for behavior change, i.e., precontemplation, contemplation, action, maintenance, and termination (18). Given the two-year duration, the study is conceptualized and designed as an early-implementation research effort. As such, long-term outcomes related to maintenance and termination are beyond the project’s timeline. Instead, the focus is on early adoption and initial implementation outcomes. We propose to follow implementation research (IR) principles and methods to develop the omnichannel intervention for CERC in VBDs, which will include a *formative or exploratory phase* and a *preparatory or development phase*.

### Aim

To co-develop a CERC strategy through a ground-up approach that the health system can digitally deliver to the community for control and elimination of VBDs, particularly dengue/malaria and lymphatic filariasis.

### Objectives

#### Formative phase

*Objective 1*: To assess the existing CERC landscape in terms of knowledge, attitude, practice (KAP), and risk perception of the community regarding VBD (with a focus on malaria, dengue, and LF), document barriers and facilitators in the current CERC strategy in the health system, and identify potentially effective communication channels for individuals and the community.

#### Development phase

*Objective 2*: Co-development of the omnichannel intervention and its implementation strategies for CERC in VBDs.

*Objective 3*: To evaluate the feasibility, acceptability, fidelity, and early adoption of the Omnichannel CERC intervention.

## Methods

### Study sites and target population

**Figure 1:**
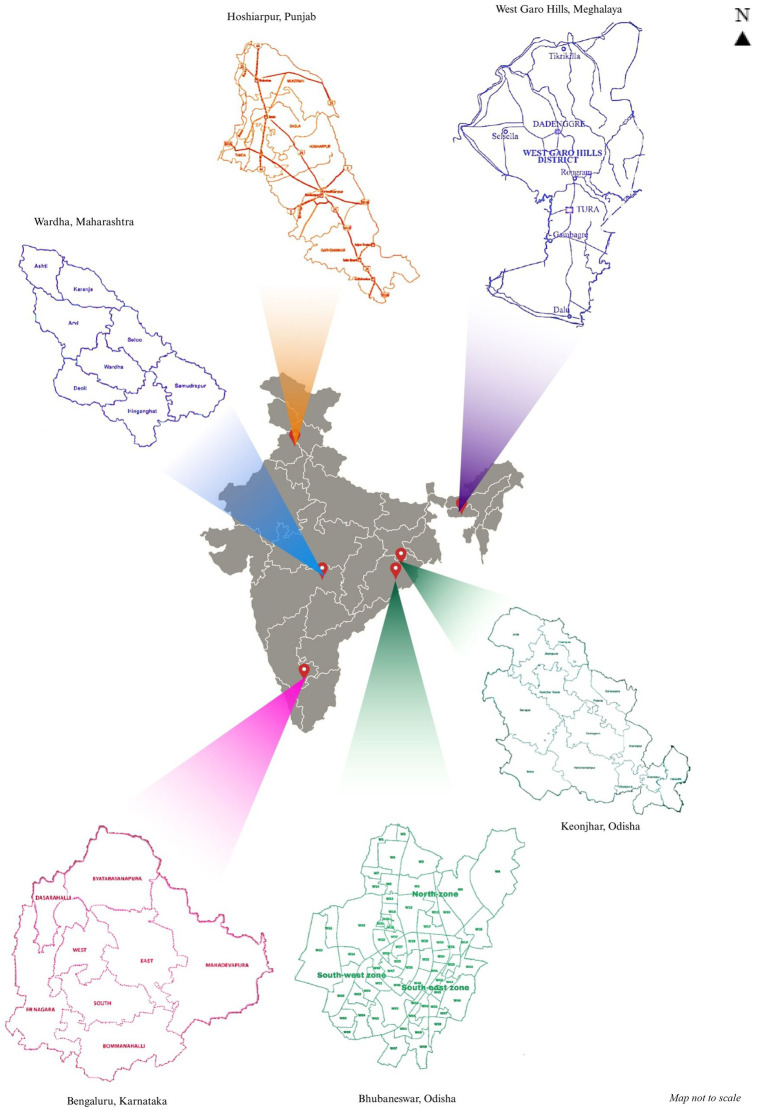
Omnivec-India study sites.

The study will be implemented at five geographically and epidemiologically distinct sites across India, located in Punjab, Maharashtra, Karnataka, Orissa, and Meghalaya (Figure 1 and Table 1) over 2 years from September 2024 to September 2026. All sites are nationally recognized, competent, academic, or research institutes, chosen to geographically represent North, South, East, West and Northeast India, based on the prevalence of VBDs of interest and the target population, i.e., urban and/or rural, underprivileged, and hard-to-reach.

**Table 1:** Study sites and populations.

#	Study site	VBD focus	Target population	Area
1	ICMR-NIP, New Delhi	Dengue	100,000	Bhunga Block, Hoshiarpur, Punjab
2	MGIMS, Sevagram	Dengue	159,877	Deoli Block, Wardha, Maharashtra
3	ICMR-RMRC, Odisha	Dengue + LF	~230,000	Ghasipura, Keonjhar (LF), Bhubaneswar (Dengue), Odisha
4	IIPH Shillong	Dengue + Malaria	100,000	Tribal population in West Garo Hills, Meghalaya
5	St. John’s Medical College, Bengaluru	Dengue	80,000	Urban under-privileged population in Bengaluru

The study area at each site was selected in consultation with local health authorities based on the prevalence of VBD and implementation logistics; i.e., the field practice area for each study site was chosen. As the study is population-based, all people residing in the area are, by default, the study population.

### Design and implementation frameworks

This is a mixed-methods implementation research (IR) study, planned over 2 years in 2 phases: a formative phase and a subsequent development phase.

**Figure 2:**
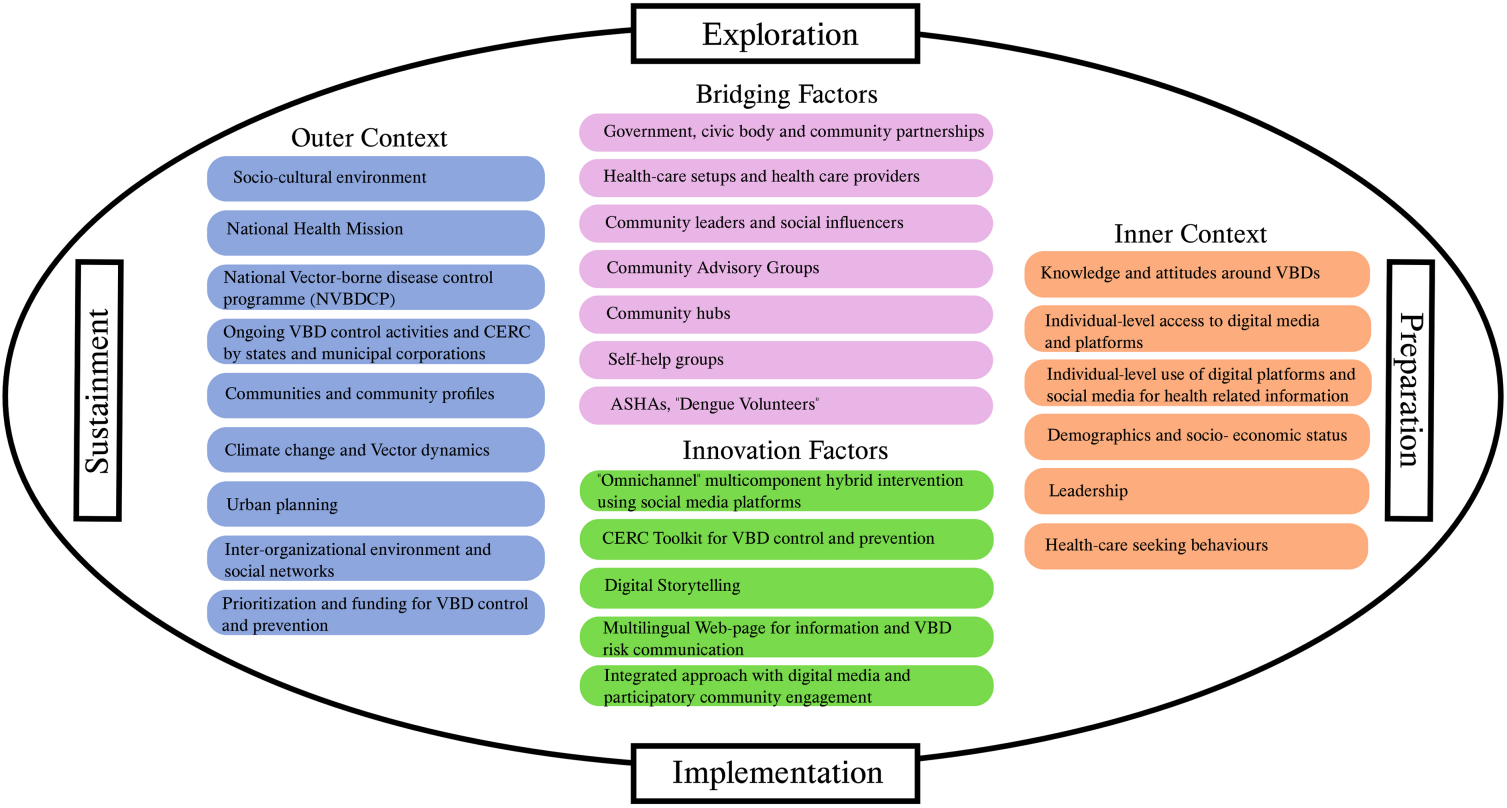
EPIS framework.

The study will use the EPIS (Explore, Prepare, Implement, Sustain) framework (29) (Figure 2) for implementation. This framework summarizes the internal and external determinants expected to influence study outcomes and the implementation of interventions. Using the EPIS framework, the study will identify the motivators and barriers for implementing the omnichannel CERC intervention (described in detail below) and enable its development and optimization. Modifications to the intervention based on feedback throughout the pilot and early implementation period will help iterate on it in parallel.

**Figure 3:**
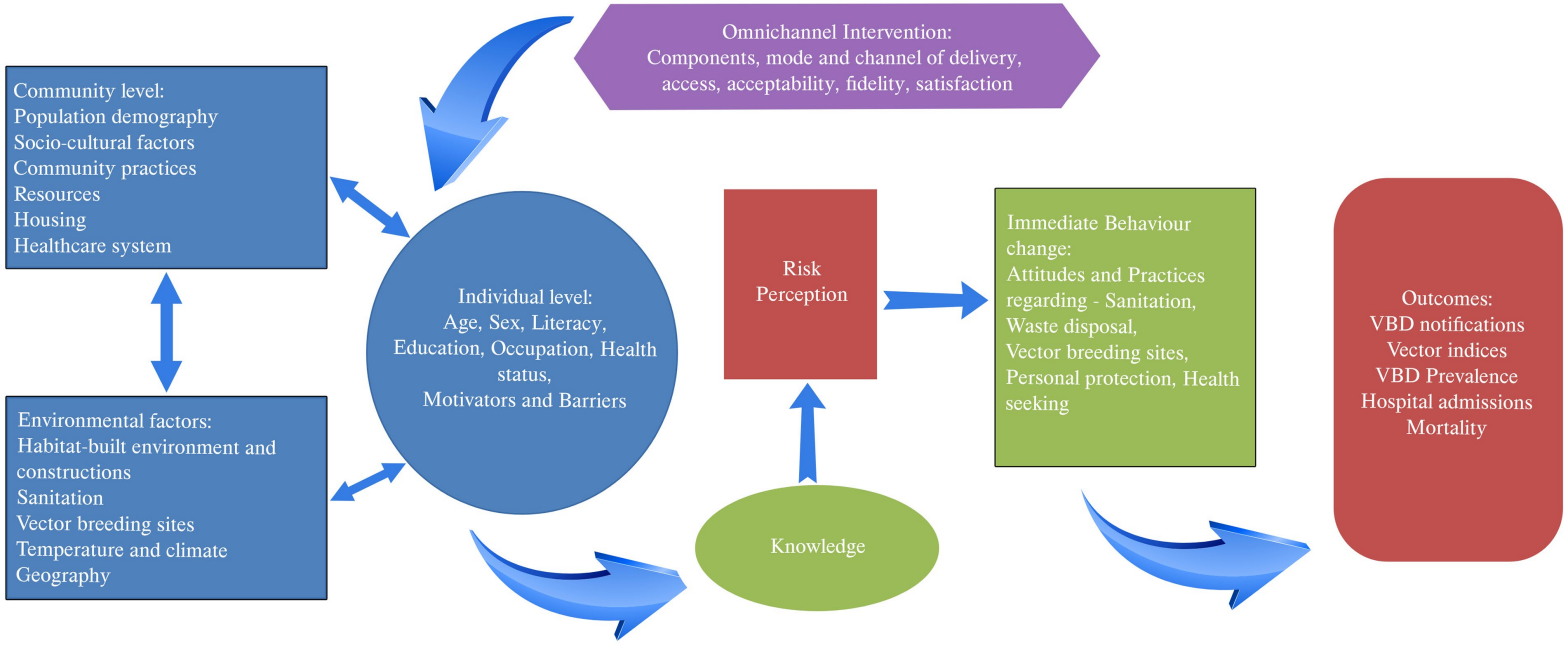
Theory of change.

In line with the EPIS framework, the theory of change that we propose suggests that factors at the environmental, community, and individual levels interact, resulting in the prevalent VBD scenario (Figure 3). The intervention, based on its accessibility, acceptability, mode of delivery, fidelity and satisfaction, will bring about behavior change through the transtheoretical model and ultimately change VBD outcomes.

The change logic model for the study (Table 2) describes the assumptions, resources, activities, enablers, outputs, and outcomes, aligned with the EPIS framework and the theory of change.

**Table 2:** The change logic model for VBD-CERC.

Assumptions	Community and health system will participate in co-designing CERC package The health system will adapt and lead delivery of omni-channel CERC package with a nudging / handholding of research team District Health System will channelize implementation through Primary health centers Community is willing to accept the intervention and change behavior for VBD prevention NVBDCP will implement the intervention if effective, across the country	
Input and resources	Finances within the community and the project available for the intervention Personnel- project staff, state and local health system personnel, anganwadi workers, school teachers, community, private practitioners, nurses, lab-technicians, pharmacists, local leaders, traditional village headmen Time for development of the intervention and its implementation Public Health system and facilities- state/district/local-CHC, U/PHC, Subcenters, health and wellness centers, anganwadis, schools, libraries, play grounds. Local self-health groups and other community groups, i.e., youth and women Private health system- clinics, private hospitals, private laboratories, pharmacies National health programs and their implementation policies and personnel. Local public works and sanitation committees Artists, singers, play-writes, dancers, story tellers Available - internet and mobile networks, TVs and local channels, radio, cinema, newspapers Transport and accessibility to and from the study areas Communication experts as well as software developers and end IT personnel Local influencers	
Activities	Formative assessment: Qualitative- mapping and identifying stakeholders, social and resource mapping Survey for KAP and intervention acceptability Focus group discussions and in-depth interviews Identifying acceptable modes of digital communication for the intervention. Identifying CAG members Identifying the first circle of communication responsible for snowballing the intervention Review of existing CERC strategies for VBD Training - staff, CAG, stakeholders, health system personnel in VBD CERC	Intervention Co-development and pilot Developing an action plan for CAG members for VBD CERC Identifying and training local hubs Health education material development for the community Website development Integrating various channels of communication into the intervention Identifying sentinels to relay misinformation. Deploying the intervention in pilot mode Obtaining feedback from the community regarding the intervention Iterating the intervention based on feedback from the community/ implementers Formation of Community hubs Assessment of change in KAP Obtaining data regarding vector breeding and VBD notifications
Enablers	Motivation, existing knowledge, education and resources, existing National Vector Borne Disease Prevention Program, Perceived and felt needs of the community, mobile penetration and use	
Output	Achievement of objectives Community resources mapped Levels of KAP identified Motivators and barriers to VBD prevention identified Preferred modes of digital communication identified CAG formed CAG action plan Contact information of first circle members for intervention deployment	Community hubs identified and set up Trained personnel IEC material developed Intervention developed and piloted Satisfaction with the intervention Acceptability of the intervention Misconceptions addressed
Outcome	Pre-post: Improvement in KAP wrt VBDs Long term: Improvement in VBD notifications Reduction in potential vector breeding sites- household and community Reduction of deaths, disability, hospital admissions, serious illness due to VBD	

As the 2-year study timeline does not support implementation and sustainability assessments, we expect to hand over the intervention to the NVBDCP for phased implementation nationwide.

### The intervention

**Figure 4:**
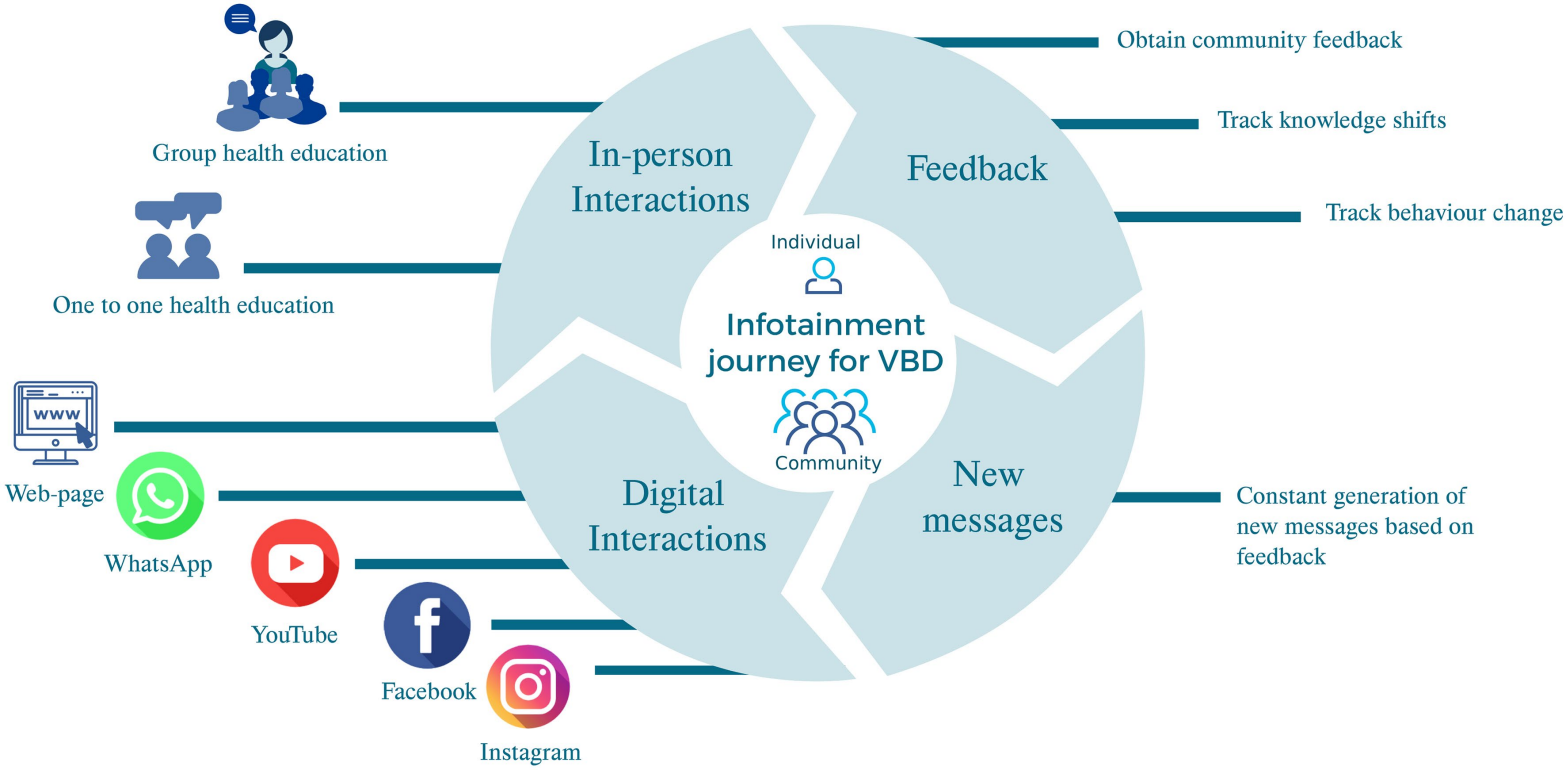
Omnichannel intervention schema.

We will use an *omnichannel digital health intervention strategy* (Figure 4) that incorporates CERC in VBDs (19). An omnichannel engagement strategy integrates multiple communication channels in a synchronized operating model (20). It leverages data and digital tools to deliver a seamless, consistent experience, focusing on the community/individual to drive the desired behavior change. The intervention will combine various social media channels and web pages with offline (in-person) supplementation of the communication. The content of the intervention will be co-developed through community engagement. The intervention will also include the constitution of a Community Advisory Group (CAG) comprising community and health system members identified through stakeholder mapping. The CAG will actively involve itself in the development, implementation and dissemination of the intervention. In addition, local hubs within community-based organizations will support dissemination of the intervention and provide offline support for implementation.

### Implementers

The project will be *co-implemented* at the district level. The implementers comprise the community, including people affected with VBDs, and the CAG (6–10 representatives from the community, local VBD hubs, and the public health system). The public health system includes urban or rural primary health centers, block authorities, and district health authorities, comprising NVBDCP officers, who are considered the overarching stakeholders. For the purposes of this IR study, the research team at each site will act as coordinators (Table 3).

**Table 3:** Levels of implementation and implementers for the VBD-CERC intervention.

Levels of implementation		Implementers role	
1	District Health Authorities- VBD Cell	Facilitate implementation of VBD CERC through the Taluk and District levels Supervise activities and review reports Provide feedback on content and activities of the intervention from the perspective of the district.	Research Team: Co-designing, development and implementation of the intervention alongside the 5 primary implementers of the intervention Obtaining feedback and iterating the intervention Evaluating feasibility, acceptability, fidelity of the intervention
2	Taluk and Block health authorities	Facilitate implementation with the Taluk or Block Supervise activities and collate reports Review and approve components of the intervention Provide feedback on implementation	
3	Primary health centers- Urban and Rural	Participate in the CAG Provide information on changing patterns of VBDs and geographic distribution of VBDs in the area	
4	CAG *(8–10 people from the community, community based organizations, local health system and VBD control program)*	Development of VBD VERC intervention Review and feedback of the VBD CERC intervention Dissemination of VBD CERC intervention Network with Health system, stakeholders and research team Support establishment of local hubs Support implementation of VBD CERC activities based on messages developed that indicate a call to action Support identification of community sentinels Identify solutions for VBD CERC sustainability Prepare and submit reports of activities to the health system	
5	Local hubs *(community based institutions that will provide a space and host in-person community engagement activities)*	Established by the CAG and will be run by volunteers from the community, identified by the CAG Support development of VBD CERC along with the CAG Relay VBD CERC information to the community Act as a nodal center in the community that collects and disseminated information related to VBDs Provides a designated space where the community can interact and obtain information on VBD prevention and control	
6	First circle *(mobile phone contacts of CAG members most likely to forward messages received)*	Report to the CAG and will be monitored by the CAG Participate in the development of VBD CERC Relay VBD related information to the community Ensure actionable items in the VBD CERC communication are implemented.	
7	Community	Participate in the development of the intervention Relay information received to others in the community Implement call to action for VBD prevention and control in the area Provide feedback regarding VBD-CERC intervention Participate in social mapping and stakeholder mapping.	

### Formative phase

The expected duration of this phase is 9 months. It will involve a situational analysis (objective 1) to inform the development of intervention.

#### Objective 1: assessing the existing CERC landscape

*Qualitative assessments* (21, 22): Transect walks and social mapping will be used to build rapport with the study population, become familiar with the study area, and map resources relevant to VBD prevention. Stakeholder mapping at the community level will involve social mapping and the use of community-drawn Venn diagrams, followed by brainstorming with selected stakeholders. Furthermore, in-depth interviews (IDI) and focus group discussions (FGD) with relevant stakeholders will be conducted to understand the demand- and supply-side challenges and the facilitators of a digital CERC-VBD prevention strategy. At each study site, we will conduct 10–15 in-depth interviews and 3–4 focus group discussions with stakeholders and the community, including those affected by VBDs, while keeping data saturation and resource constraints in mind.

*Quantitative assessments:* KAP regarding VBDs and perceptions of VBD risk will be collected at each study site using a face-validated, interviewer-administered questionnaire (Table 1).

This cross-sectional study will involve an estimated 500 participants at each study site [57% prevalence of knowledge (13), confidence limits (d) of 5%, and design effect of 1, inflated by 20% and rounded off to the nearest hundred using OpenEpi, Version 3]. The sample size was also estimated to ensure 90% power with 13% change in knowledge post-intervention. The survey will also assess the acceptability of digital technology for CERC in VBDs. Trained research assistants will identify potential participants aged ≥18 years who have resided in the study area for at least 1 year through systematic random sampling of households. The questionnaire will be administered to a consenting household member in the local language.

*Scoping review:* A simultaneous scoping review of digital and non-digital CERC strategies will indicate those with potential for use in the Indian context.

*Health system readiness for digital CERC:* A readiness assessment of the health systems for the adoption of a digital CERC strategy will involve the public health system, private or non-governmental organizations, and community-based organizations (CBOs) in the study area. The assessment will be cross-sectional and exploratory. Participants will include representatives from top, mid, and lower managerial levels, as well as staff, who will respond to a digital questionnaire via a link sent to their email or mobile phone. The questionnaire, a modified version of the Texas Christian University Organizational Readiness for Change (TCU-ORC) (23), will be used to assess motivation for change, resource adequacy, staff attributes, and organizational climate.

Outcomes of the formative phase will include KAP levels, the proportion of participants with adequate KAP, potential CERC strategies for the Indian context, the number and types of digital interventions used globally for CERC, and the levels of organizational readiness to adopt technology for CERC. These outcomes will guide intervention development.

### Development Phase

The expected duration of this phase is 15 months. It will involve the development, deployment, and evaluation of interventions (Objective 2 and 3).

#### Objective 2: co-development of the intervention and its implementation strategies

The co-development of the omnichannel intervention will involve the health system, the CAG, the community, including the people and families affected by VBDs, the media, the researchers, and Information Technology (IT) system providers, engaged either commercially or through a collaboration. The intervention will be disseminated via social media. The activities in this phase include designing the intervention, developing content, developing a capacity-building toolkit, building capacity, and establishing local hubs.

*Designing and developing the intervention:* The study proposes developing an *omnichannel*, multicomponent hybrid intervention, informed by the formative phase and in consensus with CAG. The formative phase will inform the type of communication (audio-visual, text, or voice calls), preferred content, preferred social media channels by age group, and level of interactivity. The target population’s access to mobile phones, the internet, and other digital tools will also be captured.

Community participation and workshops will enable the development of digital content for CERC to be used in the intervention. The CERC will include digitized posters, comics and art co-developed with the community. The content will also comprise digital storytelling and audiovisuals. Webpages, WhatsApp, Instagram, Facebook, or X (formerly Twitter) will host and deliver content based on target audience/community preferences. Additionally, the community will be encouraged to use digital interactive channels to post queries and clarify doubts. Calls to action (CTAs) sent to the community will encourage behavior change while enabling evaluation of the intervention, including reach, adoption, and fidelity. The CTA is a ‘digital command (prompt)’ designed to get the target audience to take a desired action, e.g., *click the arrow for more information*. The content will include vector control, the risk of acquiring VBDs, and the clinical and socio-economic consequences. The content will be adapted for offline communication, e.g., posters, flipcharts, and scripts.

Dissemination of the omnichannel intervention will be done through the CAG to their first circle of contacts (i.e., contacts on their phones who reside within the target population), who will snowball the content into the community. The information shared via digital media may be supplemented through in-person engagement with the CAG, health workers, NGOs/CBOs, schools, and the research team, especially in areas with limited internet and social media access. In-person engagement will comprise group activities, i.e., group discussions, demonstrations, street plays, child-to-child programs, and one-to-one communication at people’s homes, health facilities, or local hubs.

Capacity building for the CERC strategy will involve activities that empower the community to prevent and control VBD and address misinformation. A CERC capacity-building toolkit will be developed and used to train CAG members. The toolkit will comprise information on VBDs and their prevention, community-level prevention strategies, principles and techniques of communication, and approaches to sustaining behavior change. Capacity building will also involve establishing *community hubs* at schools, anganwadis, sub-centers, Primary Health Center, or local libraries/community cultural centers, trained to implement the CERC strategy and supported by the research team. The local hubs will also support offline communication, serving as walk-in information kiosks on VBDs.

Misinformation will be addressed through *community sentinel*s identified by the CAG. Community sentinels are community members who are likely to receive the most forwards and to forward the most messages they receive to others. The sentinels will also relay all VBD-related information they receive to the monitoring team (in this case, the project team) for verification. The messages received for verification will be classified as fact or fake and documented. For each fake message, a factual counter-message will be released.

#### Optimization and early implementation of the intervention

The intervention (model zero) will be tested and implemented over a year alongside its development, using a quasi-experimental pre-post, mixed-methods design, with the baseline set in the formative phase. The intervention will cover at least 1/4 of the overall study area in the urban setting and 5–12 contiguous villages in the rural setting, identified either through simple random sampling or based on logistical constraints. The intervention will be disseminated through the channels described earlier, and misinformation will be addressed. The CTA embedded in the disseminated information will be used to evaluate intervention coverage.

The model will be iteratively refined until an optimized version is achieved, drawing on product, user behavior, and context-based analytics that are geotagged to inform and enhance the intervention design and delivery. Community feedback will also be incorporated.

Intervention fidelity will be ensured through community sentinels and CTA. Messages that community sentinels receive will be matched with the original messages. In addition, internet metrics for access and interaction with the messages will be evaluated.

#### Objective 3: evaluation of the intervention

The primary outcome of this study is an acceptable and feasible digital omnichannel intervention for CERC in VBDs. A combination of the diffusion of innovations (DOI) theory (24), the taxonomy of implementation outcomes (25), and the RE-AIM (Reach, Effectiveness, Adoption, Implementation, and Maintenance) framework will be used to evaluate and assess the study’s outcomes (26). The intervention will be evaluated for acceptability, feasibility, early adoption, and fidelity.

*Acceptability* will be assessed based on content, complexity, and comfort with the intervention. *Feasibility* will indicate the suitability of the intervention for its intended purpose. *Early adoption* will assess community uptake and use of the intervention through calls to action, while *fidelity* will assess whether the intervention was delivered or deployed as intended.

The evaluation will also comprise a change in KAP at the end of the study period. For this, KAP will be reassessed in the study area in 500 newly identified people 6 months after the intervention is deployed from each study area, selected through systematic random sampling of households, replicating the methodology used in the formative phase. Additionally, FGDs and IDIs (as in the formative phase) will be used to obtain feedback and experiences with the intervention, with data saturation informing the sample size. CTA performance metrics, such as click rates, bounce rates, time on page, and conversion rates (the number of completions of the desired action), will be captured and analyzed. These indices will inform intervention feasibility, accessibility, acceptability, early adoption, and fidelity of the intervention.

*Long term outcome indicators* (Table 2) (i) clinical/ disease related, i.e., change in VBD notifications (i.e., number of VBDs reported per 100,000 people), change in rates of hospital admissions, serious illness, disability and mortality (disease specific mortality, and case fatality) [secondary data—public health management information system HMIS] as well as (ii) vector indices such as change in numbers of vector breeding sites (secondary data from the VBD program), are beyond the scope of the study given its 2-year timeline.

### Analysis

*Quantitative:* Frequencies, measures of central tendency, and dispersion will describe the data. Knowledge, attitude, and practice, the outcomes of the formative phase will be scored, and converted to a binary outcome based on acceptable levels of KAP, i.e., at least a 50% KAP score at the individual level. Aspects of KAP that need focus (inadequate knowledge and misinformation) will be identified for further action. Factors associated with KAP will be identified using bivariate and multivariate regression analysis. The acceptability of digital technologies and organizational readiness will also be analyzed.

Changes in KAP scores, assessed using bivariate and multivariate regression analyses, will reflect significant changes between baseline and the end of the study, as well as the factors associated with those changes.

*Qualitative:* The qualitative data will be triangulated during analysis. Narratives will be prepared for the social mapping and Venn diagrams, while FGDs and IDIs will be transcribed and translated into English for analysis. Data will be analyzed using the Framework approach for qualitative data analysis (27). The data will be coded, summarized, and abstracted. The abstracts will be categorized, and categories will be mapped under sub-themes and themes. Qualitative data from the evaluation of the development phase (at the end of the study) will be analyzed similarly.

*Patient and public involvement:* The study intervention will be co-developed and implemented with the community, including people affected with dengue in the study area, as well as the stakeholders from the public health system, NGOs, and CBOs.

### Ethics and dissemination

Ethics approval has been obtained from the Institutional Ethics Committee at each participating institution. Permissions from community gatekeepers and the health system will be sought prior to study initiation. Informed consent will be obtained for activities involving individual participation, such as the KAP study, FGDs, and IDIs.

Data will be anonymized to ensure confidentiality and password-protected, with access limited to the research teams at the study sites and the ICMR.

Findings will be shared with stakeholders through dissemination events at all levels, both at the end of the formative phase and the development phase of the study. The ICMR, the World Bank, and the NVBDCP, both at the District, State, and National levels, will receive reports and policy briefs. The findings will also be shared through national and international scientific fora and peer-reviewed publications.

## Discussion

The study aims to co-develop and evaluate early implementation outcomes of a digital CERC strategy for VBDs in India. Five study sites in north, central, south, western, and northeastern India are part of this ICMR-coordinated study. The sites are geographically, culturally, and epidemiologically diverse, especially for VBDs, although dengue is prevalent at all sites. However, given the economics and the timeframe for implementing this project, each site will focus on one (two in Odisha and Meghalaya) VBD.

We chose digital technologies for CERC in VBDs as they are pervasive, advanced, and economical in the Indian context. However, it is the seamless integration of virtual and in-person communication that makes the omnichannel approach appropriate for CERC in VBDs. Moreover, due to disparities in geography and digital coverage between study sites, for example, some study sites involve rural and tribal populations, certain forms of digital communication may pose a challenge.

Furthermore, given the prevailing socio-cultural norms, the limited awareness regarding the root cause of VBDs, and the widespread health-related misinformation (especially via social media) are probably best addressed through a digital CERC intervention in the Indian context.

We will therefore leverage the digital omnichannel marketing strategy for CERC in VBDs. The omnichannel strategy provides the end user with a seamless personalized experience, making it distinctive from a multichannel strategy (28). The intervention is dynamic and dependent on data and digital analytics generated on a day-to-day basis (10).

While the intervention’s delivery mode is predefined, we will co-design its components and content with the community and the health system, ensuring people-centeredness. The formative (preparatory) phase will therefore extensively explore knowledge, attitudes, and practices regarding VBDs, both quantitatively and qualitatively. The formation of the CAG will ensure community and healthcare system inputs during the development of the intervention, as all designs will need the CAG’s approval. Snowballing the intervention into the community through the CAG’s *first circle* of contacts and local hubs will enhance its credibility and broaden its reach. Furthermore, VBD *sentinels* within the community will validate the intervention’s reach and fidelity. They will also capture misinformation, thereby providing surveillance and quality control.

Additionally, CTA, a digital marketing tool, will not only drive the desired action (29) but also assess the reach, acceptability, early adoption, and fidelity of the intervention. CTA are prompts such as ‘*Click here, next, answer quiz, comment, share, swipe, buy now* which elicit immediate action. As this is an omnichannel intervention, we will use CTA such as ‘*meet us at the local hub or join us for a street play or movie’*, providing opportunities for offline (in-person) communication. CTA results in a desired action being successfully completed, measured as ‘*conversions’* in marketing terms. The CTA is generally built around the LIFT model comprising five components, i.e., relevance (match context and intent), clarity (should not be vague), value proposition (gains), urgency (need for immediate action), anxiety (address user concerns if action taken), and distraction (action provides the precise information sought without unnecessary elements) (30). The CTA is therefore probably the most appropriate technology within the omnichannel intervention, as it drives the desired action while quantifying it.

The strengths of the study are its multicentric design, varied VBDs addressed, and diverse populations covered, making the intervention developed comprehensive and transferable to similar contexts not only within India but also globally. The leveraging of digital media and marketing principles for CERC is likely to ensure the intervention’s uptake and sustainability. Furthermore, the mixed-methods design for both development and evaluation of outcomes will ensure the people-centredness necessary for behavior change interventions. Nevertheless, some study populations may prefer communication modes different from those conceived for this study. The intervention in these areas will reflect these preferences. While personalizing the intervention might pose a challenge, user feedback and the CTA, along with specific internet metrics, are expected to address this, making this intervention truly user-centric. Furthermore, the 2-year duration of the study limits the evaluation of change in clinical and vector indices. Given the quasi-experimental study design, controlling for external factors not adjusted for in the analysis, may be challenging.

In conclusion, a co-designed VBD-CERC intervention that ensures community-centricity is likely acceptable and sustainable within local communities. Community/people centric interventions can improve public health outcomes by driving behavior change. Such interventions also hold promise in public health emergencies such as pandemics, for creating awareness and communicating risk. Furthermore, the digital VBD-CERC intervention we propose has potential for use in other public health diseases of importance, both in India and globally.

## Omnivec study team

Abhishek Raut, Agam Jain, Arjun Jakasania, Ashwini Kalantri, Chetna Maliye, Gagandeep Singh Grover, Hitesh Kumar Jain, Jenefa Leeli Pushpam, Mary Chaithanya Yekula, Minu Rose Mani, Prakash Kumar Sahoo, Pravakar Sahu, Radhika Sharma, Rathna Kumari, Subhendu Kumar Acharya, Suklang Malngiang, Swapnil Bele, Tanuja Mishra, Tanveer Rehman.
